# Carbohydrate-Binding Mechanism of the Coagulant Lectin from *Moringa oleifera* Seeds (cMoL) Is Related to the Dimeric Protein Structure

**DOI:** 10.3390/molecules29194615

**Published:** 2024-09-29

**Authors:** Matheus Cavalcanti de Barros, Ana Patrícia Silva de Oliveira, Franciane Gonçalves dos Santos, Fabiana Aparecida Cavalcante Silva, Thais Meira Menezes, Gustavo de Miranda Seabra, Juliana Sakamoto Yoneda, Luana Cassandra Breitenbach Barroso Coelho, Maria Lígia Rodrigues Macedo, Thiago Henrique Napoleão, Thâmarah de Albuquerque Lima, Jorge Luiz Neves, Patrícia Maria Guedes Paiva

**Affiliations:** 1 Departamento de Bioquímica, Universidade Federal de Pernambuco, Recife 50670-901, PE, Brazil; matheus.cavalcantibarros@ufpe.br (M.C.d.B.); lcbbcoelho@gmail.com (L.C.B.B.C.); thiago.napoleao@ufpe.br (T.H.N.); thamarah.albuquerque@ufpe.br (T.d.A.L.); patricia.paiva@ufpe.br (P.M.G.P.); 2 Departamento de Química Fundamental, Universidade Federal de Pernambuco, Recife 50670-901, PE, Brazil; francianegs21@gmail.com (F.G.d.S.); thais.meira@ufrpe.br (T.M.M.); jorge.neves.de@gmail.com (J.L.N.); 3 Centro de Tecnologias Estratégicas do Nordeste, Recife 50740-545, PE, Brazil; fabiana.acs@gmail.com; 4 Department of Medicinal Chemistry, Center for Natural Products, Drug Discovery and Development, College of Pharmacy, University of Florida, Gainseville, FL 32611, USA; seabra@cop.ufl.edu; 5 Laboratório Nacional de Luz Síncrotron, Centro Nacional de Pesquisa em Energia e Materiais, Campinas 13083-100, SP, Brazil; juliana.yoneda@lnls.br; 6 Departamento de Tecnologia de Alimentos e da Saúde, Faculdade de Ciências Farmacêuticas, Alimentos e 22 Nutrição, Universidade Federal do Mato Grosso do Sul, Campo Grande 79070-900, MS, Brazil; ligia.macedo@ufms.br

**Keywords:** glucose-binding lectin, protein–sugar interaction, drumstick tree

## Abstract

This study characterized the binding mechanisms of the lectin cMoL (from Moringa oleifera seeds) to carbohydrates using spectroscopy and molecular dynamics (MD). The interaction with carbohydrates was studied by evaluating lectin fluorescence emission after titration with glucose or galactose (2.0–11 mM). The Stern–Volmer constant (Ksv), binding constant (Ka), Gibbs free energy (∆G), and Hill coefficient were calculated. After the urea-induced denaturation of cMoL, evaluations were performed using fluorescence spectroscopy, circular dichroism (CD), and hemagglutinating activity (HA) evaluations. The MD simulations were performed using the Amber 20 package. The decrease in Ksv revealed that cMoL interacts with carbohydrates via a static mechanism. The cMoL bound carbohydrates spontaneously (ΔG < 0) and presented a Ka on the order of 10^2^, with high selectivity for glucose. Protein–ligand complexes were stabilized by hydrogen bonds and hydrophobic interactions. The Hill parameter (h~2) indicated that the binding occurs through the cMoL dimer. The loss of HA at urea concentrations at which the fluorescence and CD spectra indicated protein monomerization confirmed these results. The MD simulations revealed that glucose bound to the large cavity formed between the monomers. In conclusion, the biotechnological application of cMoL lectin requires specific methods or media to improve its dimeric protein structure.

## 1. Introduction

*Moringa oleifera* seeds are widely used to treat drinking water for human consumption because they are a source of coagulant compounds such as lectins and organic polyelectrolytes [1,2]. Lectins are non-immune proteins capable of binding to carbohydrates and sugar-containing substances and agglutinating cells [3,4]. The ability of these proteins to recognize carbohydrates has been translated into several biological activities, with antimicrobial, insecticidal, and anticancer properties [5,6,7]. Using a gel guar column (N-galactose polymer), Santos et al. [8] purified a coagulant lectin from *M. oleifera* seeds named cMoL, which is a cationic protein of 26.5 kDa comprising 101 amino acids [9]. Insecticidal [10,11], anticancer [12], anti-inflammatory [13], coagulant [8], and antiparasitic [14] activities have been reported for cMoL. The cMoL lectin also prolongs the time required for blood coagulation, activated partial thromboplastin time, and prothrombin time [9]. In another study, cMoL demonstrated potential as a biosensor for monitoring contaminating dyes in samples [15]. Previous studies have shown that the biological activities of cMoL are associated with the ability to specifically recognize galactose; however, the mechanisms underlying this interaction remain unclear [8,15].

Carbohydrate-binding sites are directly affected by the lectin oligomerization state [16]. Therefore, characterizing the relationship between oligomerization and lectin–carbohydrate interactions is essential from a biotechnological perspective. Adjustment of the medium to obtain optimum activity in the dimeric form is essential for the development of a concanavalin A lectin-based biosensor to detect and quantify microorganisms [17]. Dimerization greatly improved the anti-HIV activity of actinohivin (an actinomycete-derived lectin) by increasing the number of binding pockets to the high mannose-type glycans of HIV-1 [18]. In seed lectin of *Dolichos biflorus*, C-terminal truncation of half of the subunits of the tetramer and dimer is essential for carbohydrate-binding activity, and only the untruncated subunits recognize carbohydrates [19]. The oligomeric state of the *Cratylia mollis* seed lectin (CRAMOLL 1) modulates protein activity by affecting its sugar-binding capacity [20].

Lectins recognize various glycans through distinct mechanisms and with different degrees of affinity [21]. Consequently, characterizing lectin–ligand interactions is crucial for optimizing the exploration of the biotechnological potential of these proteins [22]. Spectroscopic techniques are employed to characterize protein–ligand interactions, once subunit association and substrate binding promote changes in the emission spectra because of aromatic amino acid dislocation [23,24]. Molecular quenching consists of protein fluorescence suppression resulting from the interaction of the ligand with the protein [23]. For example, the mechanisms of the interaction between EgviL (a lectin derived from *Egletes viscosa*) and glucose or galactose were characterized through fluorescence quenching analysis [25]. In addition to computational tools, spectroscopy has been widely used to analyze lectin interactions with carbohydrates and glycoconjugates [26,27,28].

Understanding the interaction with carbohydrates is an essential step in finding the best conditions to explore the biotechnological potential of cMoL lectin. Therefore, in the present study, we evaluated the mechanisms involved in the interactions of cMoL with galactose and glucose using spectroscopic techniques and computational molecular dynamics simulations.

## 2. Results and Discussion

**Figure 1:**
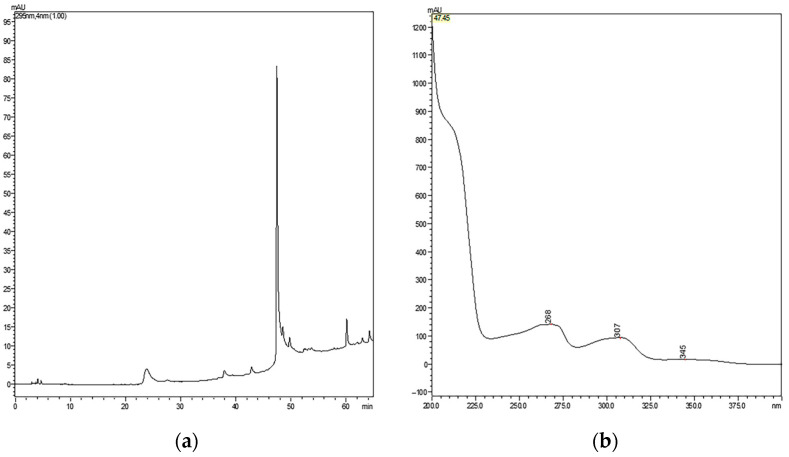
Chromatographic profile of cMoL using a C18 column in an HPLC system. (**a**) cMoL profile obtained by HPLC (0–65 min) at 280 nm. (**b**) Scan spectra corresponding to the peak.

First, the purity of cMoL was verified using the HPLC system. The cMoL profile obtained showed a high single peak at 47.452 min in the protein scanning spectra (Figure 1). The retention time observed in this study differs from the 25 min reported by Luz et al. [9] due to the employment of different columns. Specifically, this study utilized a C_18_ column, whereas the study conducted by Luz et al. [9] used a C_4_ column. Upon confirmation of cMoL purification, molecular quenching studies were subsequently performed.

**Figure 2:**
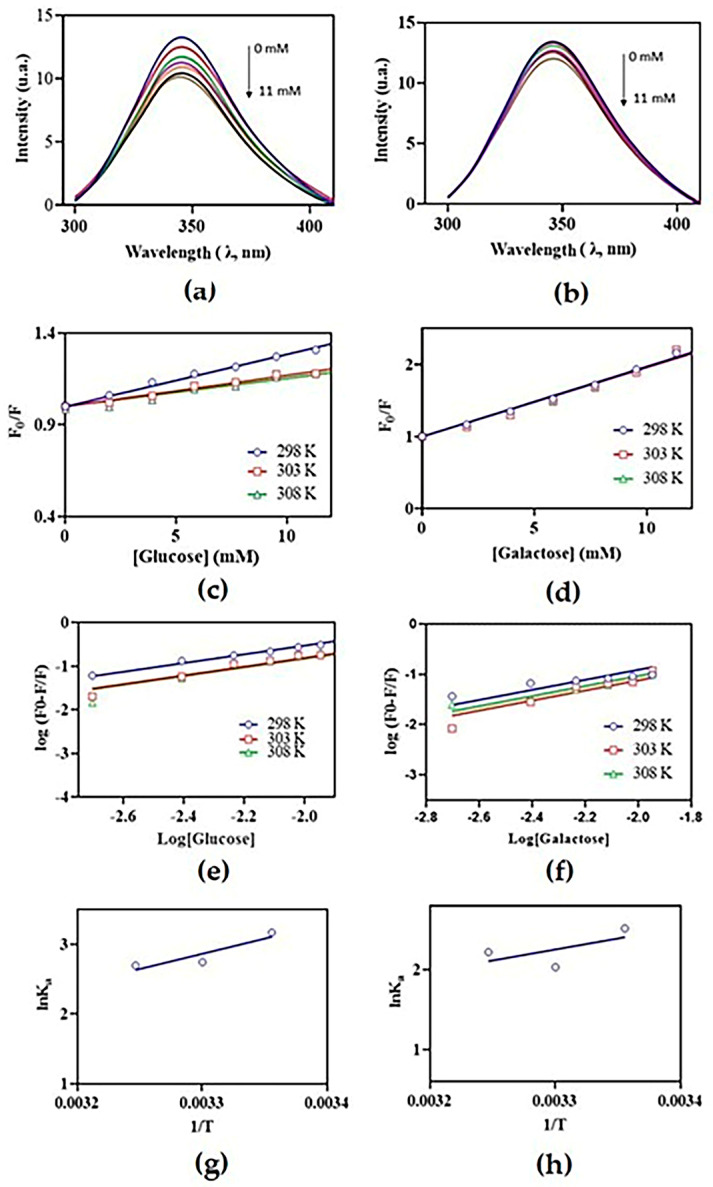
Fluorescence suppression spectra of cMoL with glucose (**a**) and galactose (**b**). Graphs obtained through the Stern–Volmer equation at temperatures of 298, 303, and 308 K for cMoL–glucose (**c**) and cMoL–galactose (**d**). Affinity constants graphs for cMoL–glucose (**e**) and cMoL–galactose (**f**). Graphs of thermodynamic parameters obtained from the Van ’t Hoff equation for cMoL–glucose (**g**) and cMoL–galactose (**h**).

A previous study evaluating the HA inhibition of cMoL by carbohydrates demonstrated that glucose and galactose were the most efficient inhibitors [8]. Therefore, quenching experiments using fluorescence spectroscopy were performed by varying the temperature and glucose or galactose concentration at a fixed protein concentration to elucidate the interaction mechanisms between cMoL and these carbohydrates. Molecular quenching refers to the phenomenon of fluorescence suppression caused by the interaction between a ligand (quencher) and a protein (fluorophore). This effect is described by the Stern–Volmer equation, which is used to determine the quenching constant (K_sv_) [23]. The quenching phenomenon is classified as static or dynamic, depending on changes in K_sv_ values at different temperatures. The suppression of the Stern–Volmer mechanism is either dynamic, because an increase in temperature favors collisions, resulting in a gradual increase in K_sv_, or static when the increase in temperature decreases the K_sv_ values, owing to the formation of non-fluorescent complexes in the ground state [22,29]. The cMoL spectral profile obtained showed a decrease (quenching) in fluorescence in the presence of increasing concentrations of carbohydrates (suppressors) (Figure 2a,b). The linear relationship between the relative intensities (F0/F) and molar concentrations indicated the formation of cMoL–monosaccharide complexes. The decrease in the suppression constant (K_sv_) with increasing temperature (Figure 2c,d) confirmed that the static mechanism is involved in this interaction. cMoL–monosaccharide complexes were static, since the suppression constant (K_sv_) decreased with increasing temperature. Consistent with these results, static quenching was also observed in the α-D-glucose/mannose specific lectin from *Litchi chinensis* seeds [30]. Conversely, the interaction mechanism between lectins from the floral capitula of *Egletes viscosa* (EgviL) and carbohydrates (glucose and galactose) was dynamic, as an increase in K_sv_ values was observed as the temperature increased [25].

A modified Stern–Volmer equation is employed to calculate the binding constant (K_a_), which is used in the Van ’t Hoff equation to determine the thermodynamic parameters associated with the interaction, such as enthalpy (ΔH), entropy (ΔS), and Gibbs free energy (ΔG) [31]. The affinity of the complexes was determined from the K_a_, which showed values on the order of 10^2^ (Table 1) and higher cMoL selectivity for glucose than for galactose. K_a_ values of 10^2^ or 10^3^ are expected for lectins since these proteins typically bind reversibly and specifically to carbohydrates through weak interactions, such as hydrophobic interactions and hydrogen bonds [11]. Comparatively, EgviL lectin showed K_a_ values on the order of 10^3^ for glucose and galactose [25]. The recombinant Jacalin, a lectin from *Artocarpus integrifolia*, displays affinity for methyl-α-galactose with a K_a_ on the order of 10^2^ [32]. Interestingly, the data obtained revealed a higher affinity of cMoL for glucose, despite earlier studies highlighting this lectin primarily as a galactose-binding protein [8,15].

The K_a_ values were used to determine the thermodynamic parameters using the Van ’t Hoff equation (Figure 2g,h). For both carbohydrates, ΔH > 0 and ΔS < 0 suggest that the complex is stabilized by hydrogen bonds and hydrophobic interaction. The negative Gibbs free energy values indicated that the interactions occurred spontaneously, favoring the formation of the complex (Table 1). For both glucose and galactose, ΔH > 0 and ΔS < 0 suggest that the cMoL—monosaccharide complex is stabilized by hydrogen bonds and hydrophobic interaction [31]. The hydrogen bonds are formed at the carbohydrate-recognizing site because of the presence of hydroxyl groups in the carbohydrate, and the hydrophobic interactions are of the CH-π type, occurring because of the stacked aromatic rings, with the hydrophobic surface formed by the adjacent CH groups of the carbohydrates [33]. The negative Gibbs free energy values indicated that the interactions occurred spontaneously, favoring the formation of the complex. The intrinsic fluorescence quenching of *Fusarium solani* lectin revealed that the binding of the protein with galactose is enthalpically driven and exothermic in nature (ΔH < 0), occurring spontaneously (ΔG < 0) [34]. EgviL showed spontaneous interactions for glucose and galactose, binding the first carbohydrate through electrostatic interactions (ΔH < 0, ΔS > 0, and ΔG < 0) and the second by hydrophobic forces (ΔH < 0, ΔS > 0, and ΔG < 0) [25].

Data from molecular quenching experiments were used to calculate the Hill coefficient. Hill plots have been used to investigate cooperativity in a variety of ligand–protein systems, where positive cooperativity gives rise to plots with slopes greater than 1.0, whereas negative cooperativity results in plots with slopes less than 1.0 [35]. The Hill parameters obtained for cMoL interactions with glucose and galactose were ~2 (Figure S1). The Hill parameter (h~2) indicated that binding events occur through a cMoL dimer, differing from those obtained for ordinary binding, where h is unitary. *Pseudomonas aeruginosa* PA-I lectin interacted with the fluorescent hydrophobic probe, 2-(p-toluidinyl) naphthalene sulfonic acid, with a Hill coefficient of 3.8 ± 0.3, showing that 2-(p-toluidinyl) naphthalene sulfonic acid probably bound to four high affinity hydrophobic sites per PA-I tetramer [36]. Dectin-1, a C-type lectin-like receptor that recognizes β(1–3)-glucans, such as laminarin, bound to the ligand cooperatively, with a Hill coefficient of ~3 [37].

The cooperativity data indicated by the Hill coefficient stimulated the conduction of an experimental model to evaluate whether the dimeric structure of cMoL is the conformation responsible for carbohydrate interaction. Therefore, an experimental model was conducted using urea at different concentrations as a denaturing agent. Chemical denaturants such as urea are widely used in protein denaturation studies due to their effectiveness in disrupting non-covalent interactions that stabilize the native protein conformation [38]. Urea-induced denaturation occurs through direct hydrogen-bonding interactions with proteins or indirect changes in the water structure, which helps to reveal the protein unfolding process [39,40]. Since urea disrupts the noncovalent interactions that maintain the oligomeric structure of proteins (e.g., dimers and tetramers), the evaluation of the structural and functional effects of this denaturing agent on cMoL can support the hypothesis of cooperativity suggested by the Hill parameter. For example, conformational analysis of champedak galactose-binding (CGB) lectin under different urea concentrations, monitored with far-ultraviolet circular dichroism (far-UV CD) and tryptophan fluorescence, revealed that CGB lectin displayed a two-step transition with three states. The first transition corresponded to the transformation of the tetramer into a monomer (starting at ∼2.0 M urea and ended at ∼4.5 M urea), and the second transition reflected the unfolding of the monomer (started at ∼5.75 M and ∼7.5 M urea) [22].

**Figure 3:**
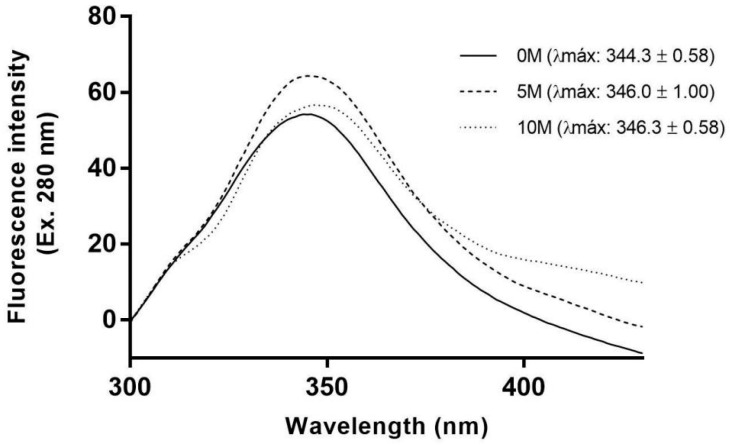
Tryptophan fluorescence spectra (Ex: 280 nm) of the cMoL lectin (mM) at different urea concentrations (0.0, 5.0, and 10.0 M) at 25 °C.

The intrinsic fluorescence of cMoL (Figure 3) increased significantly (*p* < 0.1) when the lectin was exposed to 5 M urea at a maximum fluorescence (λmax) of 346.3 ± 1.00 nm and a maximum intensity (Imax) of 64.5 ± 0.17 in relation to the untreated lectin (λmax = 344.3 ± 0.58; Imax = 53.82 ± 0.41). Notably, λmax was significantly dislocated to the red region (346.3 ± 0.58) when cMoL was exposed to 10 M urea (Imax = 56.11 ± 0.26). Urea-induced denaturation of tetrameric concanavalin A (ConA) follows a three-state mechanism involving the native tetramer, a structured monomer, and a fully denatured state. The initial stage of ConA denaturation, transitioning from tetramer to monomer, was characterized spectroscopically by an increase in fluorescence emission [41]. When exposed to 5 M urea, cMoL transitioned from the dimeric to the monomeric state, as indicated by the increase in fluorescence emission. Further exposure to 10 M urea led to a completely unfolded state, as indicated by a red shift in the fluorescence spectrum and a loss of fluorescence intensity due to exposure of aromatic amino acids residues (Figure 3). These results showed that cMoL denaturation induced by urea occurred similarly to that of ConA through monomer formation at 5 M urea, with subsequent denaturation at 10 M urea [41].

**Figure 4:**
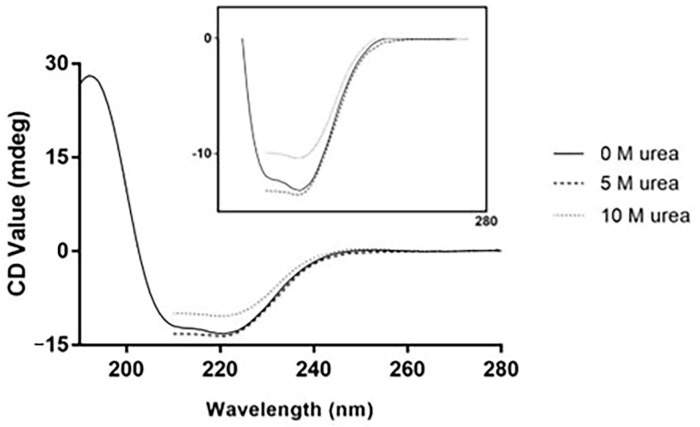
Far-UV circular dichroism spectra of the cMoL lectin showing the native, intermediate (treatment with 5 M urea), and completely denatured (treatment with 10 M urea) states at 25 °C.

The far-UV CD spectra of the cMoL lectin (Figure 4) confirmed that monomerization occurred with 5 M urea, represented by an increase in CD_218_ nm. The results obtained for cMoL were similar to those observed for the CGB lectin. The evaluation of the far-UV CD spectrum was also used to assess conformational changes in the CGB lectin exposed to 5 or 9 M urea [22].

**Figure 5:**
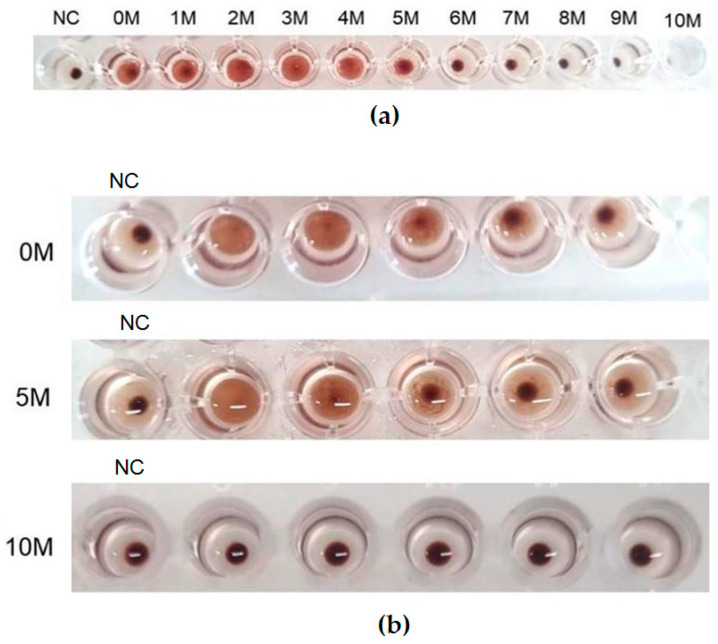
Effect of urea on cMoL hemagglutinating activity (HA). (**a**) HA of the cMoL lectin in different urea concentrations (1.0–10.0 M). Negative control (NC): erythrocytes incubated with 10.0 M urea. (**b**) HA of the serially diluted cMoL lectin at absence (0.0 M) or presence of urea at 5.0 and 10.0 M. Negative control (NC): erythrocytes incubated in absence (0 M) or presence of urea at 5.0 or 10.0 M.

A hemagglutination assay (HA) was performed to determine the activity of the lectin in the presence of urea, since treatment with 5 M urea induced the transition of cMoL from the dimeric to monomeric form. Hemagglutination results from the interaction of lectins with carbohydrates present on the erythrocyte membrane, leading to the formation of an agglutination mesh that prevents the precipitation of these cells [42]. Figure 5 shows that the treatment of cMoL with 5 M urea resulted in a partial loss of HA, showing that lectin monomerization results in the loss of the carbohydrate-binding ability. HA was not detected after the protein was completely denatured with 10 M urea. The simultaneous treatment of lectins with urea, followed by the assessment of hemagglutinating activity, has been employed in several studies to investigate lectin denaturation [25,43,44]. The results for the controls, treated by incubating only urea (5 or 10 M) with erythrocytes (Figure 5b), revealed that these concentrations did not promote erythrocyte lysis, assuring that the phenomenon observed was agglutination by cMoL. Conducting the lectin assay in the presence of urea is crucial, as removing the denaturing agent does not ensure the preservation of the protein’s oligomeric state and may lead to renaturation [45].

**Figure 6:**
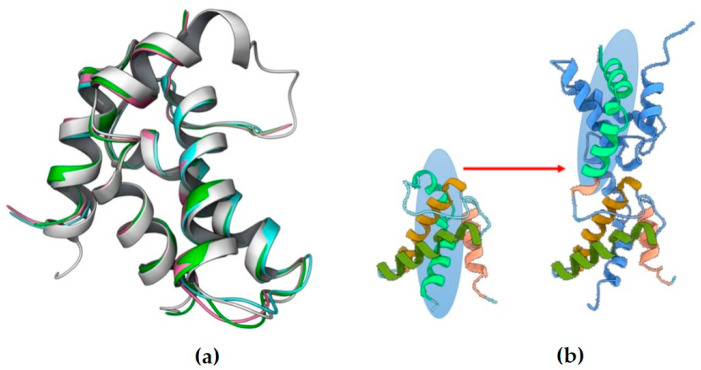
Predicted structures of the cMoL lectin. (**a**) Comparison of the predicted structure of cMoL lectin with other structures available in the Protein Data Bank (grey: cMoL (AlphaFold); green: 6JV0; purple: 5DOM; cyan: 6S3F). (**b**) Structures for the cMoL lectin monomer and dimer highlighting the movement of helix 4 (helices 1–4 of monomer 1 are shown in salmon, light brown, dark green, and light green, respectively; monomer 2 in the dimer is shown in blue).

In silico experiments involving molecular dynamics (MD) provide us with insights into lectin binding with carbohydrates, and the data can be corroborated with fluorescence quenching experiments [25]. The cMoL lectin shares high similarity with other proteins extracted from *M. oleifera* (Table 2). As shown in Figure 1, AlphaFold reliably reproduced the hypothesized structure. The predicted local distance difference test (pLDDT) values, a measure of prediction confidence, indicated high confidence in the generated structures (Figure S2). The cMoL monomer consisted of four alpha helices (residues 2–15, 19–37, 42–58, and 74–99), joined by short-loop hinges. Helices 1 and 2 formed a V-shaped structure, and helices 3 and 4 inserted in the space between the two, above and below the plane formed by helices 1–2. During dimer formation, helix 4 of each monomer was placed at the corresponding position in the other monomer (Figure 6). Monomer association in a dimeric or tetrameric structure maintained by noncovalent interactions is common for lectins in solution [19,46].

**Figure 7:**
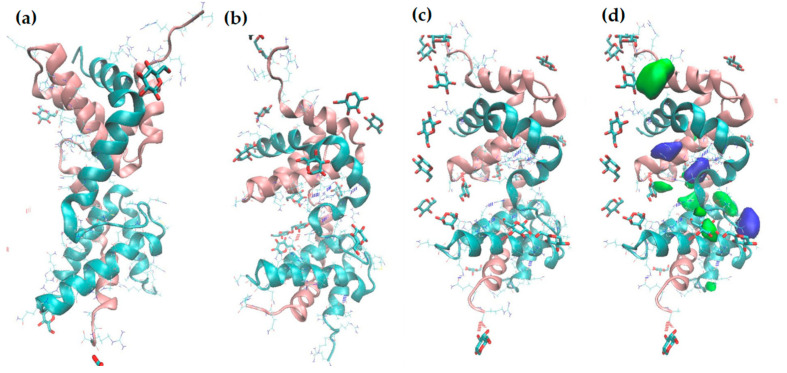
Snapshots from the MD simulation of the first replica: (**a**) initial structure, (**b**) after 150 ns, (**c**) final structure, after 500 ns. (**d**) The same structure as in (C), superimposed with the volumetric map, representing the occupancy by glucose molecules; blue: α-D-glucose; green: β-D-glucose.

AlphaFold was used to predict dimer structures as the initial point for MD simulations. Owing to the high affinity for glucose shown by the quenching experiment, MD simulations were performed in triplicate using this carbohydrate. The MD simulations showed that, during the first 200 ns of the simulation, the protein changed shape as helices 2 and 3 approached helices 3 and 2 of the other monomer, stacked on top of each other, and glucose molecules accumulated in the space between them. This structure was maintained throughout the simulation (Figure 7 and Figures S3 and S4). To locate probable interaction sites between glucose and cMoL, the VolMap tool (version 1.1.) in Visual Molecular Dynamics was used to plot the density distribution of glucose molecules in proximity to the protein. The results showed that the glucose molecules tended to be inserted into the space between the monomers in the large cavity formed between helices 2 and 3. Dimeric and tetrameric associations can be important to form cavities or carbohydrate-recognition domains, which are vital to lectin–carbohydrate binding and/or the biological activity of these proteins [50]. As observed in the quenching experiment, the interaction between cMoL and glucose was stabilized by hydrogen bonds, and the most persistent interactions found through MD are listed in Supplementary Table S1.

## 3. Materials and Methods

### 3.1. Lectin Purification

*Moringa oleifera* seeds were collected from the city of Arara, Paraíba, Brazil, with authorization (no. 72.024) from the Instituto Chico Mendes de Conservação da Biodiversidade (ICMBio) of the Brazilian Ministry of Environment. The cMoL lectin was purified as described by Santos et al. [8]. Extraction was performed through homogenization of the seed powder (10 g) in 0.15 M NaCl (100 mL) for 6 h at 28 °C. After filtration through gauze, a protein-enriched fraction was obtained by adding ammonium sulfate (60% saturation) for 4 h. The precipitate was recovered, dialyzed for 4 h to remove the salt, applied to a gel guar column equilibrated with 0.15 M NaCl, and eluted with 1.0 M NaCl. The absorbance of the eluate from the chromatographic column was measured at 280 nm. After elution, the samples were dialyzed against water to remove the eluent, lyophilized, and stored in a freezer until further analysis.

### 3.2. High Performance Liquid Chromatography (HPLC)

HPLC analyses were carried out using a Shimadzu Prominence LC-20AT device with a diode array detector (SPDM20), an SIL-20AC automatic injector, a CTO-20A oven, and a DGU-20A5 degasser. Chromatographic separation was performed with a Luna C-18 column (25 cm × 4.6 mm × 5 µm, Phenomenex, Torrance, CA, USA). After dilution in Milli-Q water (5 mg/mL), cMoL was filtered through a 20 µm filter. A total of 20 µL of the sample was injected, and chromatography was performed for 65 min at 30 °C at a flow rate of 1 mL/min. The column was equilibrated with solvent A [0.1% (*v*/*v*) trifluoroacetic acid in H_2_O] and eluted using solvent B (90% acetonitrile in 0.1% trifluoroacetic acid) in a linear gradient, where B = 5% at *t* = 0 min, B = 5% at *t* = 5 min, B = 100% at *t* = 60 min, and B = 0% at *t* = 65 min. The elution profiles were monitored at 280 nm.

### 3.3. Molecular Quenching

Fluorescence emission spectra in the range of 300–400 nm were recorded using a Jasco FP-6300 spectrofluorometer in a quartz cuvette (10 × 10 mm light path) at temperatures of 298, 303, and 308 K, with excitation at 280 nm. The excitation and emission wavelengths were fixed at 5 nm. The cMoL solution (1.4 mM) in water was titrated (20 to 120 µL) with 0.2 M glucose or galactose solutions prepared in sterile Milli-Q water. After the measurements, the Stern–Volmer constants (K_sv_) were calculated using Equation (1) [25], as follows:

(1) $$\frac{F0}{F}=1+K_{SV}\left[Q\right]$$

where F_0_ is the fluorescence intensity of lectin in the absence of carbohydrates, F is the fluorescence intensity in the presence of carbohydrates, and [Q] is the quencher (suppressor) concentration. Subsequently, the binding constants (K_a_) were calculated using a modified Stern–Volmer Equation (2), where n is the number of binding sites, as follows:

(2) $$log\left(F\right)=log\left[Ka\right]+nlog\left[Q\right]$$

From the binding constants, the thermodynamic parameters were determine using the Van ’t Hoff Equation (3):

(3) $$lnK_{a}=-\frac{∆H}{R}.\frac{1}{T}+\frac{∆S}{R}$$

where R is the gas constant, T is the temperature (K), ΔH is the enthalpy change, and ΔS is the entropy change. The Gibbs free energies (∆G) were calculated using Equation (4) [28], as follows:

(4) $$∆G=∆H-T∆S=-RTlnK_{a}$$

To evaluate cooperativity in the cMoL–carbohydrate interaction, a specific binding model with a Hill slope was applied according to the following equation:

(5) $$\frac{F-F_{0}}{F}=\frac{{Bmax\left[Q\right]}^{h}}{{Kd}^{h}+{\left[Q\right]}^{h}}$$

where K_d_ = 1/K_a_ is the dissociation constant, and h is the Hill coefficient.

### 3.4. Urea-Induced Denaturation Experiments

#### 3.4.1. Fluorescence Spectroscopy

The conformational stability of cMoL (2.5 mM) was evaluated, with or without exposure to urea (5.0 and 10 M), for 14 h at 28 °C [25]. Fluorescence measurements in the urea-induced experiment were performed as described above, except for the emission spectral range, which was configured at 300–430 nm. After treatment, the samples were transferred to a cuvette, and the intrinsic fluorescence was measured in triplicate.

#### 3.4.2. Circular Dichroism (CD)

Circular dichroism measurements were obtained in the far-UV region at 25 °C using a Olis DSM20 spectropolarimeter at the circular dichroism beamline (CEDRO) of the Brazilian Synchrotron Light Laboratory (Campinas, Brazil). The cMoL lectin (30 µM) samples in Milli-Q water or in different urea concentrations (5.0 and 10 M) were scanned between 180 and 280 nm with three scan accumulations using quartz cylindrical cells measuring 0.2 mm in path length. The bandwidth was 1 nm, and the integration time was 1 s. In the samples treated with urea, spectra could not be obtained below 210 nm owing to the signal-to-noise ratio.

#### 3.4.3. Hemagglutinating Activity (HA)

The same conditions employed in the fluorescence spectroscopy analysis were used for the HA. First, 50 µL of the protein sample in urea (1–10 M) was added to 96-well plates, with the subsequent addition of rabbit blood (50 µL), previously fixed with glutaraldehyde. The negative control corresponded to erythrocytes incubated with 0.15 M NaCl or different urea concentrations in the absence of lectin. After incubation for 45 min at 28 °C, the precipitation of erythrocytes was observed. Subsequently, the samples exposed specifically to 0.0, 5.0, and 10 M of urea were subjected to serial two-fold protein dilution (50 μL) in 0.15 M NaCl, according to the procedures described by Paiva and Coelho [51]. The HA was defined as the highest dilution of the protein sample that promoted the full agglutination of rabbit erythrocytes. Blood collection was approved by the Ethics Committee on Animal Experimentation of the Universidade Federal de Pernambuco (Process no. 23076.033782/2015-70).

### 3.5. Molecular Dynamics (MD) Simulations

In silico analyses were conducted based on the cMoL sequence (QARRPAIQRCCQQLRNIQPRCRCPSLRQAVQLAHQQQGQVGPQQVRQMYRVASNIPAICNLQPMYCPFGQGQQQQQCRQQFLTHQRLRACQRFIRRQTQGR), published by Luz et al. [9]. Models for the cMoL lectin monomer and dimer were created using AlphaFold 2.0 [52,53]. The predicted structure of the dimer was used as the initial structure for the MD simulations. A simulation box was prepared with one copy of the dimer, 27,500 water molecules, 100 glucose molecules (36 α-D-Glu and 64 β-D-Glu), and 58 Na^+^ and 90 Cl^−^ ions, resulting in concentrations of 0.2 M glucose and 0.15 M salt. The Amber ff19SB force field [54] was used for the proteins, Glycam_06-j [55] for the sugars, and the OPC model for water [56]. The system was initially subjected to 100 minimization steps (10 steepest descent (SD) and 90 conjugate gradient (CG) steps), in which only water molecules were allowed to move, followed by 10,000 minimization steps (1000 SD + 9000 CG), in which all atoms were allowed to move. The system was then heated at constant volume to 310 K for 0.6 ns, followed by 0.4 ns at the final temperature, using a 2 fs MD step. The system was then allowed to relax for 10 ns at a constant pressure of 1 atm and a temperature of 310 K before being subjected to another 500 ns for the data production stage. The entire process was simulated in triplicate. All simulations used the Amber 20 package for biomolecular simulations [57], and the analysis was performed using CPPTRAJ released with the AmberTools 22 package, and Visual Molecular Dynamics v1.9.3 [58].

### 3.6. Statistical Analysis

The data are shown as the mean ± standard deviation. Fluorescence spectroscopy and CD graphs were plotted using GraphPad Prism 7.0 (GraphPad Software, Boston, MA, USA). The CD data were deconvoluted using the CDTooX software [59]. The normality of the fluorescence data was determined using the Shapiro–Wilk test. One-way analysis of variance (ANOVA) was performed, followed by Tukey’s post-test. Statistical significance was set at *p* ≤ 0.1.

## 4. Conclusions

The formation of cMoL–monosaccharide complexes is favored through a static suppression mechanism and negative Gibbs energies, indicating a spontaneous process. These complexes are stabilized by hydrogen bonding and hydrophobic interactions. The Hill plot, confirmed by fluorescence spectroscopy, CD, and HA, showed that the binding of cMoL with glucose and galactose occurs through a protein dimer. Molecular modeling has shown that glucose binds to large cavities formed between monomers. Finally, the biotechnological application of cMoL in the production of biosensors or as a natural coagulant must employ methods or media that favor the dimeric structure of the protein as the carbohydrate-binding property, and consequently, its biological activities are related to this oligomeric state.

## Figures and Tables

**Table 1:** Thermodynamic parameters of cMoL interactions with glucose and galactose.

Carbohydrate	T (K)	K_sv_ (10^2^ M^−1^)	K_a_ (10^2^ M^−1^)	∆H (J/mol)	∆S (J/K·mol)	∆G
Glucose	298	0.2847 ± 0.0020	0.29785 ± 0.00007	4.358	−11.51	−7.681
	303	0.1701 ± 0.0014	0.15559 ± 0.00005			−6.764
	308	0.1542 ± 0.0013	0.14825 ± 0.00007			−6.904
Galactose	298	0.9768 ± 0.0007	0.12387 ± 0.00004	2.752	−6.82	−6.371
	303	0.9610 ± 0.0009	0.07617 ± 0.00001			−5.124
	308	0.9584 ± 0.0080	0.09212 ± 0.00001			−5.686

**Table 2:** Sequence identity of cMoL compared to different *Moringa oleifera* proteins with crystal structures available in the Protein Data Bank.

PDB ID	System	Res (Å)	Identity (%)	Reference
6VJ0	Mo Chitin-binding protein	1.90	81.2	PDB
5DOM	Mo albumin	1.69	81.2	[47]
6S3F	Mo seed protein Mo-CBP3-4	1.68	81.2	[48]
2DS2	Mabinlin II	1.70	73.9	[49]

## Data Availability

The dataset is available on request from the authors.

## Supplementary Materials

The following supporting information can be downloaded at: https://www.mdpi.com/article/10.3390/molecules29194615/s1, Table S1: The 20 most persistent hydrogen bond interactions from all three simulations; Figure S1: AlphaFold structure of the cMoL monomer (left) and dimer (right), with chain A colored by pLDDT values (higher values mean higher confidence in the predicted position of residues). The values are similar for monomer 2 in the dimer; Figure S2: Root mean square deviation (RMSD) of the protein atoms during each replication.; Figure S3: Root mean square fluctuations for the residues in the dimer simulations. The bold dark line is the average of the three RMSFs. Figure S4: Root Mean Square Fluctuations for the residues in the dimer simulations. The bold dark line is the average of the three RMSFs.
