# Supplementary material for: Carbohydrate-Binding Mechanism of the Coagulant Lectin from Moringa oleifera Seeds (cMoL) Is Related to the Dimeric Protein Structure
Source: Molecules. 2024 Sep 29;29(19):4615. doi: 10.3390/molecules29194615 (PMC11477877; doi:10.3390/molecules29194615)
Supplement: Supplementary file 1 [file molecules-29-04615-s001.zip › molecules-3081915-supplementary.pdf]

Table S1. The 20 most persistent hydrogen bond interactions from all 3 simulations.

Protein	Structure	Occupancy (%)
**B:Arg_101**	C-Term	18.83
**B:Arg_4**	H1	17.79
**A:Arg_101**	C-Term	17.16
**B:Pro_24**	H1-H2 Loop	15.51
**A:Arg_96**	H4	12.46
**A:Gln_72**	H3-H4 Loop	11.06
**B:Gln_74**	H4	10.99
**A:Tyr_65**	H3-H4 Loop	10.33
**A:Phe_68**	H3-H4 Loop	9.94
**B:Gln_97**	H4	9.93
**A:Pro_67**	H3-H4 Loop	9.91
**B:Hid_34**	H1-H2 Loop	9.69
**B:Pro_5**	H1	9.56
**B:Gln_99**	H4	9.54
**A:Gln_8**	H1	9.41
**B:Gln_75**	H4	8.81
**A:Gln_97**	H4	8.73
**A:Gln_35**	H1-H2 Loop	8.59
**B:Gln_70**	H3-H4 Loop	8.52
**B:Gln_39**	H2-H3 Loop	8.5

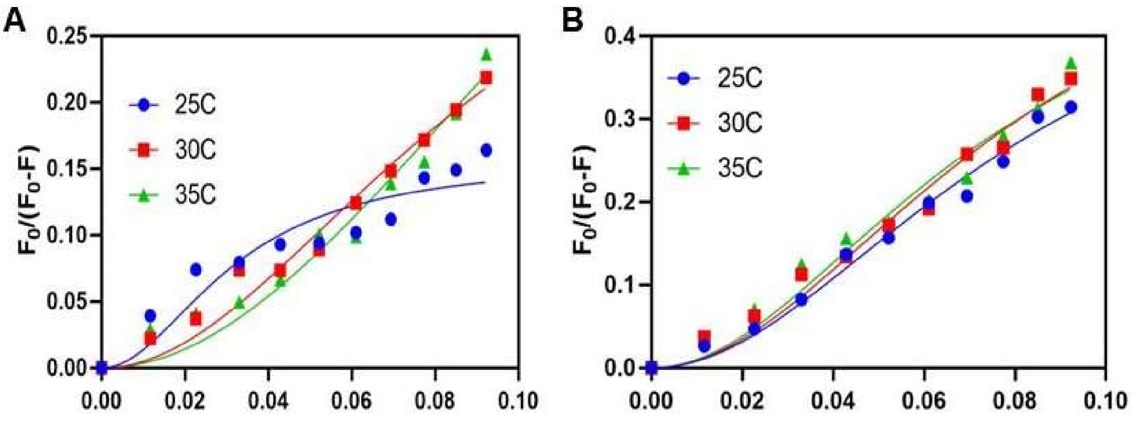

Figure S1. Hill plots for cMoL-galactose (a) and cMoL-Glucose (b) complexes.

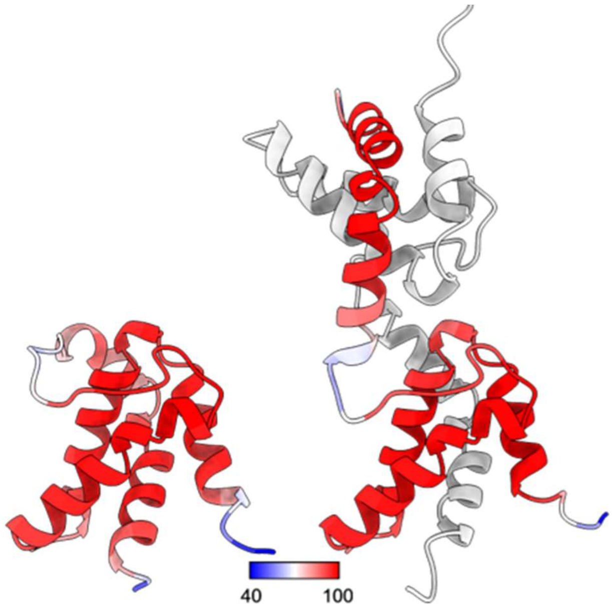

Figure S2. AlphaFold structure for the cMoL monomer (left) and dimer (right), with Chain A colored by pLDDT values (higher values mean higher confidence in the predicted position of residues). The values are similar for monomer 2 in the dimer.

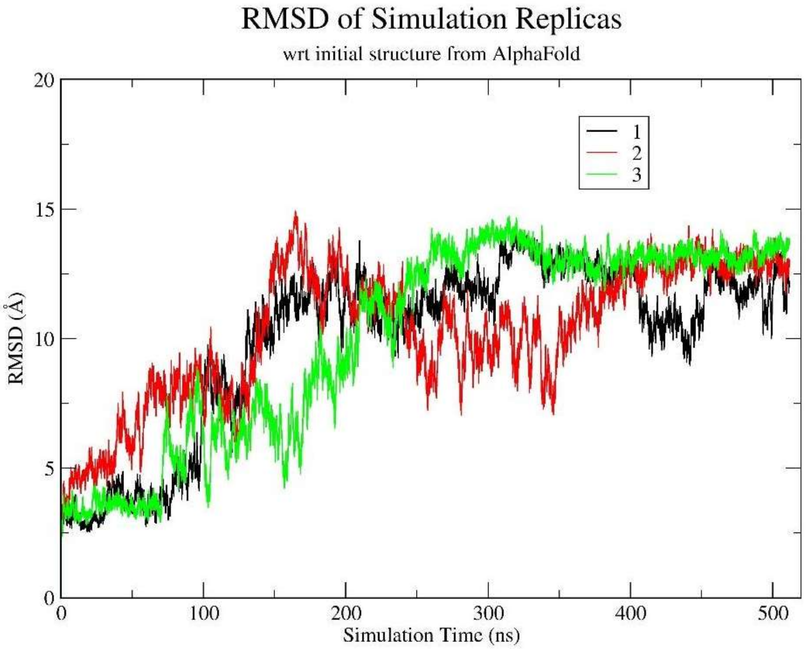

Figure S3. Root Mean Square Deviation (RMSD) of the protein atoms during each replica.

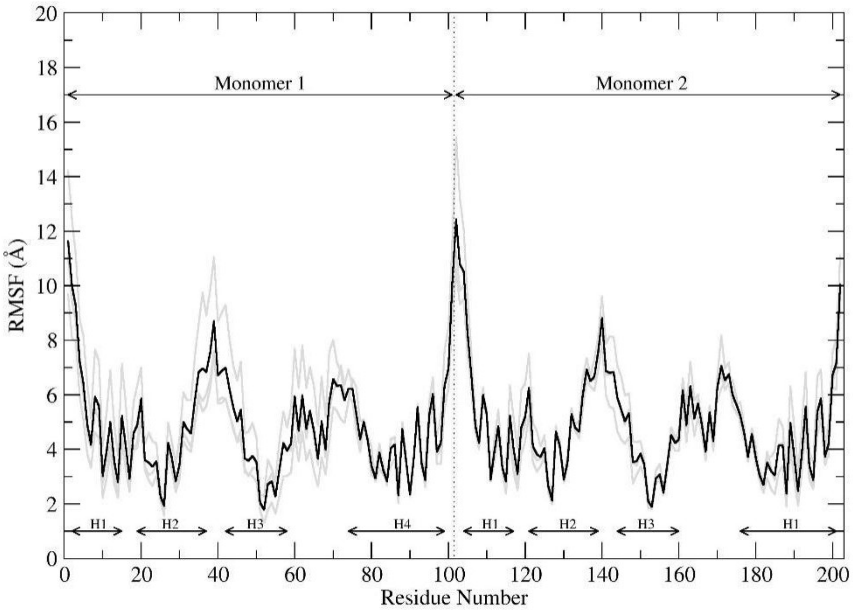

Figure S4. Root Mean Square Fluctuations for the residues in the dimer simulations. The bold dark line is the average of the three RMSFs.
